# 30-Second Chair Stand and 5-Times Sit-to-Stand Tests Are Interesting Tools for Assessing Disability and Ability to Ambulate among Patients with Multiple Sclerosis

**DOI:** 10.3390/life14060703

**Published:** 2024-05-30

**Authors:** Andrea Polidori, Mattia Malagoli, Rosario Giacalone, Giampaolo Brichetto, Margherita Monti Bragadin, Valeria Prada

**Affiliations:** 1 Scientific Research Area, Fondazione Italiana Sclerosi Multipla (FISM), 16149 Genova, Italygiacalonegiovannirosario@gmail.com (R.G.); giampaolo.brichetto@aism.it (G.B.); margherita.montibragadin@aism.it (M.M.B.); 2 Servizio Riabilitazione Liguria, Associazione Italiana Sclerosi Multipla (AISM), 16149 Genova, Italy; mattia.malagoli@aism.it

**Keywords:** multiple sclerosis, rehabilitation, outcome assessment, treatment outcome, outcome measures

## Abstract

Multiple Sclerosis (MS) is a demyelinating and chronic disease with variable neurological symptoms. There are different scales that score the level of disability, but only few papers have taken into consideration the 5-times sit-to-stand (5STS) test and the 30 s chair stand test (30CST), which are valid and easily obtainable indicators of other neurological diseases. The aim of our research is to verify the validity, reproducibility, and responsiveness of these tests. Patients afflicted with MS were enrolled in the AISM outpatient facility. The inclusion criterion was an EDSS score less than 6.5. We performed the 5STS, 30CST, and timed 25-foot walk (T25-FW) tests and recorded EDSS scores in the first evaluation. Then, we recorded the performance after 5 days (conducted by a second blind operator to ensure test–retest reproducibility), and the last evaluation was made after 12 sessions of physiotherapy. We recruited 38 patients diagnosed with MS. The results show significant data regarding validity, reproducibility, and responsiveness for both scales. The data argue in favor of adding these tests to the relevant clinical assessments. These two tests are simple, reliable, and easy to administer, and the data confirm that they can be included in the evaluation of patients with MS.

## 1. Introduction

Multiple Sclerosis (MS) is a chronic, demyelinating, and inflammatory disease resulting from an autoimmune attack against the white matter of the central nervous system [1]. It is characterized by a transient inflammation of myelin and axons which can lead to the development of large focal lesions in the white matter of the brain and in the spinal cord, causing different levels of disability, ranging from moderate to severe, followed by a partial or total recovery [2]. MS affects young adults between 20 and 40 years of age. In 0.4% to 10.5% of cases, the onset occurs in childhood (before the age of 16 years), and the prevalence ratio of women to men has increased markedly during the last few decades (2.3–3.5:1), which indicates a significant increase in MS among women but not men.

MS can be subdivided into three main phenotypes, namely, (i) relapsing–remitting; (ii) primary progressive; (iii); and secondary progressive [3,4], although a radiologically isolated syndrome and a clinically isolated syndrome have recently been described as two new disease courses [3].

All the course phenotypes exhibit great variability in presentation depending on the position and the quantity of lesions in the brain or spinal cord. MS can cause a wide range of symptoms and potentially affect every part of the body. Moreover, these symptoms are unpredictable, and each person can be affected differently. Some of the most common symptoms are fatigue, vision problems, numbness, muscle stiffness and/or weakness, pain, bladder and bowel problems, speech and swallowing disorders, and balance and walking impairments [5].

We can also recognize different clinical subtypes based on different parameters, including tremors, ataxia, visual disturbances, sensory symptoms (numbness and paresthesia), pyramidal tract findings (weakness, hyperreflexia, spasticity, and hypertonia), or spinal cord findings (paraparesis, sphincter dysfunction, and sensory levels) [5]. People with MS (pwMS) can show cognitive impairment as well [6].

Among all these symptoms, having difficulty walking is a common concern in pwMS. Usually, walking impairments are one of the early symptoms [7,8], and this dysfunction occurs at different levels, ranging from a slight and barely visible impairment except with great tiredness or in impervious sections to a significant impairment that requires the use of orthoses, aids, or walkers or wheelchairs for short stretches or most of the time. Moreover, unstable walking can be related to a higher risk of falls and balance impairment [9]; therefore, the use of a rapid test that can evaluate all these components during an assessment has become important in the daily clinical evaluation of patients.

There are several clinical scales pertaining to the assessment of walking and balance impairments among MS patients. Among the scales routinely used, there is the BERG balance scale for balance (BBS) [10], and we can find many scales for evaluating walking concerns, which differ in terms of the distances travelled and/or the administration time (the Six-Minute Timed Walking Test (6MTWT), the Two-Minute Timed Walking Test (2MTWT), the Timed 25-Foot Walk Test (T25-FW), and the Timed Up-and-Go (TUG) test) [11].

Some of these tests often require longer administration time. In fact, the BERG balance scale, for example, takes 15–20 min, as reported in the literature [10]; the 6MTWT and 2MTWT, which are largely used for evaluating MS, are also considered long tests [12].

Moreover, these tests could expose patients to the risk of falling.

The TUG test, for example, does not require much time, and its performance safety has been demonstrated [13,14]. However, especially for patients with balance problems or significant motor issues, careful and scrupulous supervision is necessary during the walking and turning phases. The TUG, in fact, is a very effective outcome measure [14], but it requires the use of many complex motor skills (standing, walking, turning, walking again, and sitting down), which are often affected in neuromotor disorders.

For this reason, all these tests require continuous supervision by the operator and extra attention for patients with a moderate or severe motor disease [15]. Even though the risk of falling during the assessment is minimal, it can still induce anxiety, stress, and fatigue in patients with moderate walking dysfunctions [16].

Over the years, the sit-to-stand movement has been a topic of significant study because it is a daily activity attracting significant clinical interest. Indeed, it requires good motor control and stability [17]. For this reason, tests involving the ability to sit and then stand can detect and predict impairments in more complex activities, such as walking, since they assess the basic characteristics related to walking [18].

The Thirty-Second Chair Stand Test (30CST) and Five-Times Sit-to-Stand (5STS) test are two tests that evaluate the sit-to-stand movement [8,19]. They do not require a walking task during their administration, and their administration time is short.

In the literature, there are many works showing that the 30CST and 5STS are both effective indicators for assessing the risk of falling for patients with balance disorders and evaluating the loss of lower-limb strength or walking impairments [20]. The populations represented in the literature are elderly people, people with chronic obstructive pulmonary disease (COPD) [21], stroke patients [22,23], women with breast cancer [21], and individuals afflicted by various other neurological diseases, such as Parkinson’s disease [13,24].

Reference values of the 30CST and 5STS for healthy individuals are present in the literature [25,26]. The psychometric parameters of the 30CST and 5thS for pwMS have been investigated by various authors over the past three years, and their results demonstrated excellent interrater reproducibility and test–retest reproducibility [27].

In addition, they significantly correlate with the most common outcome measures for assessing the level of disability of patients with MS [28,29]. A recent study showed a significant positive correlation between 5STS and the Brief International Cognitive Assessment for MS (BICAMS) and the Modified Fatigue Impact Scale (MFIS), demonstrating that the sit-to-stand movement can be related to cognitive impairments and fatigue [30].

However, no studies in the literature also evaluated the responsiveness of these two tests, and the correlation with T25-FW is still uncertain; in fact, only one paper has investigated the correlation between the 30CST and T25-FW [31], while no studies have investigated the correlation between the T25-FW and the 5TSTS.

The aim of this study is to provide a complete verification of the validity, reproducibility, and responsiveness of the 5STS and the 30CST for pwMS. In fact, taking into account the traits of these patients and the frequent involvement of the walking and balance area, it would be important to consider these practical tests in routine clinical practice in order to obtain more information in only a few minutes and ensure full safety for the patients.

## 2. Materials and Methods

### 2.1. Design of the Study

This is an observational study. At T0, we selected the patients who met the inclusion criteria and performed the evaluation of the scales reported below. After 12 sessions of motor rehabilitation, we repeated the same assessment at T1. Motor rehabilitation was tailored and individualized for every patient. Following a medical visit, a physiatrist provided a rehabilitation recommendation for the physical therapist. All patients involved completed a physiotherapy regimen that included motor exercises for muscle recruitment, balance exercises, and stretching exercises.

### 2.2. Inclusion and Exclusion Criteria

We only included patients belonging to our AISM clinic in Genoa who had a prescription for 12 neuromotor rehabilitation sessions including balance and walking training. The inclusion criteria were (i) diagnosis of Multiple Sclerosis with any form of disease between relapse and remission (RR), either secondary progressive (SP) or primary progressive (PP); (ii) Expanded Disability Status scale (EDSS) score ≤ 6.5 (this means that we only enrolled patients with residual walking abilities); and (iii) a prescribed rehabilitation cycle of 12 sessions of neuromotor physical therapy. The exclusion criteria were (i) EDSS score ≥ 6.5 (this means that we excluded non-ambulatory individuals or those partially restricted to a wheelchair) and (ii) acute relapse within three months before starting the rehabilitation cycle. If an acute relapse occurred during the rehabilitation cycle, the pwMS was considered a dropout.

### 2.3. Generalities

In the first session, the operator collected the demographics and clinical characteristics of each patient by completing a case report form (CRF). The demographic characteristics included gender and age, while the clinical information comprised the duration of the disease, the phenotype of the disease (RR, SP, PP), the EDSS score, use of aids (distinguished as the use of one support, two supports, or none), and use of orthoses (distinguished as those currently in use or the lack of orthosis use). During the initial visit, each patient was asked to consent to the study’s conditions and sign an informed consent form.

### 2.4. Scales

We performed a correlation analysis of 30CST and 5thS with EDSS and T25-FW, considered the gold standard for evaluating walking difficulties and levels of disability in MS.

EDSS is based on an examination by a neurologist, and it is a clinical parameter included in a patient’s medical report [32]. This scale is useful for defining the severity of a disease suffered by a patient on a scale from 0 to 10. T25-FW was conducted with the use of an aid/orthosis if the patient regularly wore one, and the assessment was performed as described by Kalinowski et al. [33], based on the National MS Society Guidelines.

The 30CST and 5STS were administered as recommended by Zhang et al. [21], and two stopwatches were used to record the scores. If a patient could not perform the test, he/she was assigned a score of 0 in the 30CST and 250 s in the 5STS test. We followed the guidelines by using a standard chair that was leaned against a wall and had a height of 48 cm. Each patient started the test in a standardized sitting position with their feet on the ground and their upper limbs folded across their chest. An operator first explained the task to the patients, and then the patient was asked to stand up and sit down as quickly as he/she could in 30 s. Each patient performed the tests barefoot and without orthoses. The operator counted down from 30 s with one stopwatch and recorded how much time a patient needed to perform 5 sit-to-stand movements with the other stopwatch.

### 2.5. Procedure

Each enrolled patient started a standard physical therapy cycle (with 12 sessions) in the outpatient facility of the AISM rehabilitation center of Genoa (Italy). At T0, during the first session, we collected the generalities of the patients and their signed informed consent forms, and then we administered 25-FW, 30CST, and 5STS. Five days later, a different blind operator administered the 30CST and 5STS with the same modalities to check the inter-operator reproducibility. Then, after 12 sessions, the patients repeated the full battery of tests at T1.

Our local ethics committee (the Ethics Committee of the Policlinico IRCCS San Martino of Genova, Italy) approved this study, granting it the approval number 023REG2014.

### 2.6. Statistics

Mean, range, and standard deviation (SD) were calculated for all the considered parameters characterizing the considered population. Pearson’s coefficient was calculated to determine the correlation between the considered tests and the other outcome measures. Responsiveness between T0 and T1 was calculated with a paired *t*-test for non-parametric samples (Wilcoxon test). Reproducibility was described using the interclass correlation coefficient (ICC) and Cronbach’s alpha coefficient. All the statistics were considered significant when *p* < 0.05. In the graph, * indicates *p* < 0.05; ** indicates *p* < 0.01; *** indicates *p* < 0.001; and **** indicates *p* < 0.0001.

## 3. Results

We collected data on 38 patients (Table 1). The mean age of the whole population was 55.0 ± 11.29, with a range of 30–74; the sample comprised 24 females (63%) and 14 males (37%). The mean duration of the disease was 15 ± 13 years (range 1–53 years), and the mean EDSS score was 5.75 ± 1.06 (range 1.5–6.5).

The predominant MS form was RR (*n* = 25, 63%), followed by SP (*n* = 10, 26.3%) and PP (*n* = 3, 7.7%). Twelve patients regularly used an aid (31%), six of whom used one support, while the other six used two supports. In addition, seven patients used an orthosis (18%). All of the 38 patients had performed the full battery of tests at T0 (Table 2). A total of 37 patients performed the re-test after 5 days, and 32 patients completed the full physiotherapy program, since 6 patients interrupted their physical therapy sessions before T1; hence, the responsiveness analysis was based on 32 samples.

### 3.1. Validity of 30CST and 5STS

**Figure 1:**
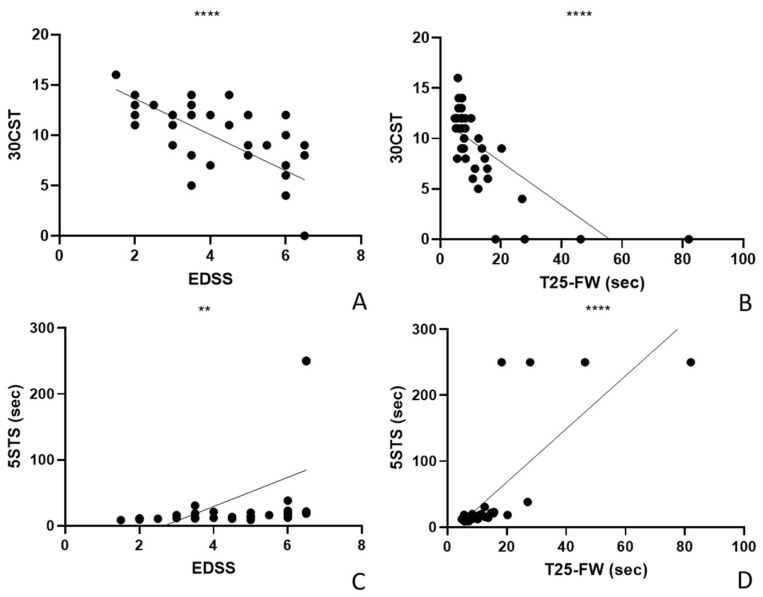
Validity of sit-to-stand tests. (**A**) 30CST shows a significant negative correlation with EDSS. (**B**) 30CST shows a significant negative correlation with T25-FW. (**C**). 5STS correlates significantly with EDSS. (**D**) 5STS correlates significantly with T25-FW. 30CST: 30 s chair stand test; 5STS: 5-Times Sit To Stand test; T25-FW: Timed 25-Foot Walk Test; EDSS: Expanded Disability Status Scale (EDSS). In the graph, * indicates *p* < 0.05; ** indicates *p* < 0.01; *** indicates *p* < 0.001; and **** indicates *p* < 0.0001.

We correlated the parameters of the 30CST and 5STS recorded at T0 with the parameters of the EDSS and the T25-FW recorded at T0 to test their validity (Figure 1). The 30CST showed a significant negative correlation with EDSS (r Pearson −0.6778, r^2^ 0.4593, *p* < 0.0001, *n* = 38, Figure 1A) and T25-FW (r Pearson −0.7204, r^2^ 0.5190, *p* < 0.0001, *n* = 38, Figure 1B). The 5STS correlated significantly with EDSS (r Pearson 0.4771, r^2^ 0.2276, *p* = 0.0025, *n* = 38, Figure 1C) and T25-FW (r Pearson 0.7749, r^2^ 0.6005, *p* < 0.0001, *n* = 38, Figure 1D).

### 3.2. Reproducibility of the 30CST and 5STS

We compared the measurements of two blind operators, and the results are shown in Table 3. The ICC was 0.983 (*n* = 37), with a confidence interval between 0.966 and 0.991; Cronbach’s alpha was 0.984 (*n* = 37) for the 30CST. Similar results were obtained for the 5STS; specifically, the ICC was 1.00 (*n* = 37), with a confidence interval between 0.999 and 1.000, while Cronbach’s alpha was 1.00 (*n* = 37).

### 3.3. Responsiveness of the 30CST and 5STS

We report the responsiveness of the 30CST and 5STS, comparing the parameters recorded at the baseline to the scores obtained after the 12 rehabilitation sessions had been conducted. Moreover, we recorded the performance of the T25-FW because we know that this is a responsive and reliable assessment.

**Figure 2:**
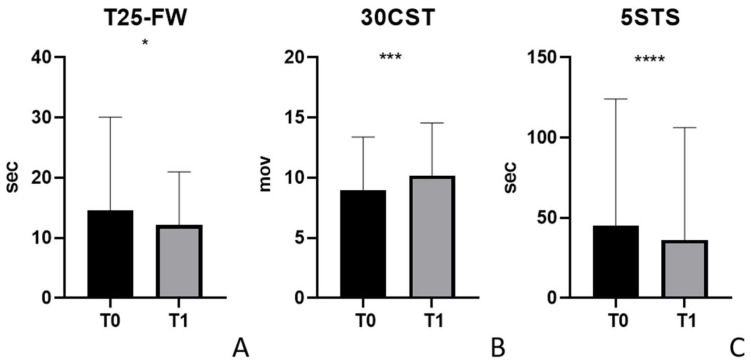
Responsiveness of T25-FW, 30CST, and 5STS. T0 refers to the first physiotherapy session; T1 refers to the 12th physiotherapy session. (**A**) T25-FW showed a significant difference (*p* = 0.039) between T0 and T1. (**B**) 30CST was significantly different (*p* = 0.0001) in T0 and in T1. (**C**) The difference between T0 of the 5STS and T1 was significant (*p* < 0.0001). 30CST: 30 s chair stand test, 5STS: 5-Times Sit-to-Stand test; T25-FW: Timed 25-Foot Walk Test; sec: seconds; mov: movements. In the graph, * indicates *p* < 0.05; ** indicates *p* < 0.01; *** indicates *p* < 0.001; and **** indicates *p* < 0.0001.

T25-FW showed a significant difference (*p* = 0.039; median of differences: −0.23; *n* = 32) between T0 (mean score: 14.60 ± 15.46 s; range: 5.5–82.00 s; *n* = 32) and T1 (mean score: 12.14 ± 8.83 s; range: 5.6–42.30 s; *n* = 32 (Figure 2A)).

For the 30CST, the difference between T0 (mean score: 9 ± 4.39; range: 0–16; *n* = 32) and T1 (mean value: 10 ± 4.38; range: 0–16; *n* = 32) was significant (*p* = 0.0001, median of differences equal to 1.000, *n* = 32 (Figure 2B)). Furthermore, the differences between T0 of the 5STS (median score: 44.05 ± 77.91 s; range: 8.5–250 s; *n* = 32) and T1 (median score: 36.02 ± 70.18 s; range 8.73–250 s) were significant (*p* < 0.0001; median differences −1.905; *n* = 32).

## 4. Discussion

The purpose of this study is to emphasize the significance of employing sit-to-stand tests since these tests have been largely studied and standardized and many papers report that there is a correlation between this movement and the quality of walking and balance.

The mean age of our sample was 55 ± 11.29 years, while the reported mean age for this disease is slightly younger. This could be explained by the fact that we only enrolled patients coming to our outpatient facility for physiotherapy treatments. In addition, we only considered patients with medium/high levels of disability (the EDSS score of our population was 5.75 ± 1.06), and this could even be influenced by the age of the patients. The female/male ratio was 2 to 1, which is in accordance with the epidemiologic data [34,35].

RR was predominant form of MS in our population, and it is also the most common form globally [35,36].

The mean EDSS score of the pwMS recruited in this paper was higher than the scores for samples in similar published works [27,30,31]; nevertheless, it is possible to observe that patients requiring rehabilitation at our center may have had higher EDSS scores compared to other populations selected for research. Our belief is that an impaired population can highlight the effects of rehabilitation in a more effective manner.

The average T25-FW score was 12.98 ± 14.08 s, which is in accordance with the average T25-FW score observed for pwMS with comparable disabilities in our sample [32]. The correlation with T25-FW also emphasized that the 30CST and 5STS can be used to safely assess walking impairments, and this is in accordance with the findings of other authors, who also correlated the two scales with 6TWT and TUG [14,27,30,31]. Furthermore, this is the first work to correlate 5STS with T25-FW.

During our assessments, we did not observe any falls or loss of balance. This information indicates that patients with medium/high disability levels may find the 30CST and 5STS safer. However, we only chose ambulant people (EDSS ≤ 6.5) to verify if these two tests can describe the level of disability of walking pwMS, which is a large range. The strong correlation between the two tests and the EDSS score confirms our hypothesis. However, it might be intriguing to limit the range of disabilities and examine more similar levels of disability in the future. It is worth considering EDSS scores > 6.5 for individuals who are not able to walk long distances but can still perform sit-to-stand movements with supervision. In fact, there are few scales that assess lower-limb function among non-ambulatory patients, despite the significance of this parameter in establishing the functional independence of a pwMS.

The high inter-operator reproducibility demonstrated that both tests are easy to administer and easily reproducible. Furthermore, they can be executed in different environments, such as at home, since both tests do not require a large space to be executed.

The great responsiveness that the two tests have shown leads to another consideration. T25-FW also showed the effectiveness of rehabilitation for these patients, but the results for the 30CST and 5STS were more significant, showing a better sensitivity to changes compared to the T25-FW. This means that sit-to-stand movement is related to the ability to walk (T25-FW demonstrated that ambulation was improved after 12 sessions). Moreover, it could be more sensitive to detecting improvement. This factor plays a crucial role when selecting tests as part of patient evaluation.

Since the sit-to-stand movement requires many neurophysiological strategies, and since MS is highly heterogeneous, it might be interesting to find out which range of symptoms can be better assessed using the 30CST and 5STS [37].

As demonstrated by Bowser et al. in 2015 [19], among pwMS, standing up is influenced by lower-limb force, spasticity, trunk control, and changes in one’s center of gravity (COG) [19]. Therefore, the goal for the future is to prove that there is a stronger correlation between the 30SCST and 5STS and the most common assessment tools for balance, lower-extremity muscle strength, and fatigue, especially with respect to the 30CST. Indeed, in the literature, the 5STS has been studied to a greater extent than the 30CST and correlated with the most common assessment tools [27,29].

This could be interesting not only with respect to highlighting the effectiveness of 30CST and 5STS as evaluation scales but also for considering the standing-up movement as a functional movement for rehab. Tulipani et al. [38], through using wearable inertial sensors, claimed that the 5STS can discriminate the risk of falls for pwMS [38]; so, it could be interesting to verify if better performance in standing up and in the sitting down could improve the safety and balance of patients.

In the literature, many papers describe the biomechanics of the sit-to-stand movement in relation to normal controls and pathological diseases [17], and the modelling of a normal sit-to-stand movement is very important for understanding the neurophysiological mechanisms underlying the ability to enter the standing position since humans are the only animals capable of bipedal walking. Furthermore, to date, there has only been one paper describing the results of a movement analysis conducted on pwMS [19], while no neurophysiological or biomechanical modelling has been conducted for this category of patients. How MS lesions can affect one’s ability to enter the standing position and then walk could be interesting. Moreover, finding different pattern categories, since patients are so variable, would be useful with respect to suggesting which rehabilitation is better in terms of improving standing and walking.

Another interesting point is that these two tests are easy to reproduce, and they require only a chair and a stopwatch. In light of the new knowledge gained after the COVID-19 pandemic, there is a need for assessments that are easy to perform, even remotely [39].

A remote assessment must be easily controllable by the specialist online, and, at the same time, it must be easy for the patient to perform and safe. Different guidelines for remote assessment are available in the literature [39]. Furthermore, the supervision and experience of caregivers are of fundamental importance, but they may not be sufficient if we ask an individual to perform a complex task. Moreover, for remote assessments, the availability of the instruments and spaces required is also important. For example, the 6MWT is a difficult test to reproduce in the home of a patient due to space issues (in many countries around the world, taking into consideration normal residential homes, having a corridor of 30 m is often impossible) and because patients with walking issues are at risk of falling.

A sit-to-stand test, on the other hand, is quick, easy to teach to a caregiver, safe, and only requires a chair.

Surely, these advantages could also apply to some hospitals where it is not possible to have a dedicated standardized corridor for walking tests and when there is not much time to dedicate to the assessment of a patient.

Certainly, there is currently a multitude of extremely effective scales for evaluating pwMS, and professionals can choose which ones to use depending on their selected objective, the dysfunction they want to highlight, and/or the type of responsiveness needed. Furthermore, the preferred scales in research are often different from those chosen in clinics, as in clinical practice, time is often short, and spaces are not optimal. In order to choose the best solution, in light of the results of our work, it is always important to use complementary evaluation scales, for example, associating a walking test with a sit-to-stand test.

In fact, choosing a simpler test for patients with moderate walking, balance, or even cognitive impairment will help to provide a more objective and less stressful and/or fatiguing assessment for patients.

In order to deepen research on this topic, we will surely need a larger and more homogeneous sample, as the deficiency of these aspects is a limitation of our study. Another limitation of the present work is the lack of a standardized physiotherapy program for each patient, which could have led to a difference in the results in the last session. However, most of our patients responded well to the treatment, improving not only their performance in the 30CST and 5STS but also in the T25-FW, attesting to the efficacy of their rehabilitation.

In conclusion, the 30CST and 5STS should be used in the evaluation of pwMS because they are two valid tests that can evaluate levels of disability and walking concerns. Furthermore, the 30CST and 5STS are reliable, rapid, easy for patients to perform, and safe compared to the most common measures for assessing MS.

## Figures and Tables

**Table 1:** Demographic and clinical characteristics of the sample.

	pwMS (*n* = 38)
Age (mean ± SD)	55 ± 11.29 years
Age (range)	30–74
Disease duration (mean ± SD)	15 ± 13
Disease duration (range)	1–53
Forms	25 RR, 10 SP, 3 PP
EDSS (mean ± SD)	5.75 ± 1.06
EDSS (range)	1.5–6.5
Use of aids	26 none, 6 one support, 6 double support
Use of orthosis	31 none, 7 current use

SD: standard deviation, RR: relapse–remission, SP: secondary progressive, PP: primary progressive, and EDSS: expanded disability status scale.

**Table 2:** Clinical outcomes at the baseline (T0).

	pwMS (*n* = 38)	
	Mean	Range
T25-FW score	12.98 ± 14.08 s	5.2–82 s
30CST score	9.18 ± 4.17	0–16
5STS score	39.98 ± 73.24 s	8.5–250 s

pwMS: persons with Multiple Sclerosis, T25-FW: timed 25-foot walk test, 30CST: 30 s chair stand test, 5STS: 5-Times Sit-to-Stand Test.

**Table 3:** Reproducibility parameters of the 30CST and 5STS.

	Crombach’s Alpha	95% CI		ICC
		Upper Limit	Lower Limit	
30CST	0.98	0.97	0.99	0.98
5STS	1.00	0.99	1.00	1.00

30CST: 30 s chair stand test, 5STS: 5-Times Sit-to-Stand test; CI: class interval; ICC: interclass correlation coefficient.

## Data Availability

The data that support the findings of this study are available from the corresponding author, VP, upon reasonable request.
